# Suitability of smoking cessation support from social and community service organizations: perspectives of Dutch clients

**DOI:** 10.1093/heapro/daae141

**Published:** 2024-10-24

**Authors:** Judith E M Visser, Judith Burger, Andrea D Rozema, Anton E Kunst, Mirte A G Kuipers

**Affiliations:** Department of Public and Occupational Health, Amsterdam Public Health Research Institute, Amsterdam UMC, University of Amsterdam, Van der Boechorststraat 7, 1081 BT Amsterdam, the Netherlands; Department of Public and Occupational Health, Amsterdam Public Health Research Institute, Amsterdam UMC, University of Amsterdam, Van der Boechorststraat 7, 1081 BT Amsterdam, the Netherlands; Tranzo Scientific Center for Care and Wellbeing, Tilburg School of Social and Behavioral Sciences, Tilburg University, Professor Cobbenhagenlaan 125, 5037 DB Tilburg, the Netherlands; Department of Public and Occupational Health, Amsterdam Public Health Research Institute, Amsterdam UMC, University of Amsterdam, Van der Boechorststraat 7, 1081 BT Amsterdam, the Netherlands; Department of Public and Occupational Health, Amsterdam Public Health Research Institute, Amsterdam UMC, University of Amsterdam, Van der Boechorststraat 7, 1081 BT Amsterdam, the Netherlands

**Keywords:** tobacco smoking, inequalities, community health promotion, disease prevention, perspectives

## Abstract

Social and community service organizations (SCSOs) may be a promising new environment to more successfully reach people with a lower socioeconomic position (SEP) for smoking cessation support. However, studies that investigate clients’ perspectives of the suitability of SCSOs as a setting to discuss smoking are scarce. This study aimed to (i) investigate the suitability of smoking cessation support provided by SCSOs, according to people with a low SEP, and (ii) explore their reasons for considering it suitable or unsuitable. Semi-structured interviews were conducted with 19 individuals with a low SEP who smoked regularly (*N* = 14) or had smoked regularly (*N* = 5). They have been in contact with SCSOs in a specific neighborhood in Amsterdam. Data were analyzed using a thematic approach. Participants generally considered SCSOs as suitable for providing smoking cessation support, as professionals are involved, build a relationship of trust, and offer personalized and holistic support. SCSOs are located nearby and familiar, they provide support in both group and individual settings and might offer additional supportive (group)activities. A number of participants expressed doubts about the waiting time for support, the fact that the needed support might exceed professionals’ expertise, and the lack of aftercare. SCSOs can be an additional opportunity for providing smoking cessation support that aligns with the circumstances of lower SEP people. To harness the potential, smoking cessation could be integrated into education programs and training among professionals could be promoted. Policy changes within and outside SCSOs would be needed.

**Contribution to Health Promotion**

- Support from social and community service organizations can be an important opportunity for people with lower socioeconomic positions in addition to standard smoking care.
- Social and community service organizations may be perceived as more accessible compared to standard smoking care.
- Professionals from social and community service organizations could be able to address both the smoking behavior as well as the underlying problems of clients’ smoking behavior.
- Clients have doubts about the capability of social and community service organizations and the professionals working within these organizations to provide effective support.
- To harness the potential of social and community service organizations, smoking cessation could be integrated into education programs and training among professionals could be promoted.

## BACKGROUND

Smoking is the leading preventable cause of premature mortality and socioeconomic health disparities in Europe (WHO, 2021). Smoking prevalence rates differ between socioeconomic positions (SEPs), as smoking is more prevalent and persistent among people with a lower SEP compared to a higher SEP (Reid *et al*., 2010; Hiscock *et al*., 2012). The higher smoking prevalence in people with a lower SEP can partly be attributed to a lower quit success rate even though their quit attempt rates are similar to those in higher SEP groups (Kotz and West, 2009; Hiscock *et al*., 2012). Smoking inequalities are a growing concern and countries acknowledge that addressing these differences in quit success is important to reduce health inequalities (Kotz and West, 2009; Hitchman *et al*., 2014).

Van Wijk *et al*. (2019) demonstrate, using the access to health care model, that people from lower SEP groups encounter many barriers in seeking and receiving professional smoking cessation support. For example, they may experience that local support is unavailable, that there is a lack of attention to their life circumstances, and that the support is insufficiently intensive or flexible (van Wijk *et al*., 2019). Previous literature reviews also emphasize that healthcare professionals experience challenges in reaching people from lower SEP groups, keeping them involved, and effectively supporting them to quit smoking (Hiscock *et al*., 2012; Twyman *et al*., 2014). For example, healthcare professionals may experience a lack of time to provide support, and some may lack the confidence and knowledge needed to assist patients in quitting smoking. In order to reduce inequality in quit success rates, Van Wijk *et al*. (2019) recommend improving access to effective cessation support for low SEP groups by providing support to overcome barriers at the various stages that an individual has to go through to actually receive the needed care. These stages involve identifying healthcare needs, seeking and reaching healthcare services, obtaining healthcare services and actually being offered services that fit healthcare needs (Levesque *et al*., 2013; van Wijk *et al*., 2019).

Evidence suggests that social and community service organizations (SCSOs) may be a promising new community-based environment, alongside healthcare, to reach and support people with a low SEP to quit smoking (Bryant *et al*., 2011, 2012). These are governmental or non-governmental organizations that offer services and facilities related to work, social participation, self-reliance, well-being and youth, with the primary goal of enhancing the quality of life of their clients and enabling them to fully participate in society (Jansen, 2018; Visser *et al*., 2024). For example, a study conducted in Australia indicates that SCSO professionals have regular contact and existing relationships with people who smoke from lower SEP groups, are well-suited to provide tailored support and are able to also address other issues faced by clients that may hinder smoking cessation (Bryant *et al*., 2011, 2012). In addition, a study conducted in the Netherlands shows that the trust between these professionals and clients facilitates the discussion of sensitive topics (Visser *et al*., 2024). Furthermore, SCSO professionals are trained to interact professionally, preventing clients from feeling pressured or judged. However, even if SCSOs may be a promising environment to reach and support this group, the perspectives of clients are crucial in exploring whether SCSOs should play an active role in smoking cessation support.

A feasibility and acceptability trial conducted in Australia showed that clients found smoking cessation support from SCSOs (i.e. assessing willingness to quit, providing advice to quit and providing access to free nicotine replacement therapy (NRT)) acceptable and helpful (Bryant *et al*., 2012). In the same country, a study found that the majority of clients reported a strong desire for support and encouragement to quit (Bryant *et al*., 2010). The opportunity to receive support from staff at the SCSO to quit smoking alongside the support already provided was viewed positively (Bryant *et al*., 2010).

While the acceptability of support among clients from SCSOs has been investigated in Australia (Bryant *et al*., 2010, 2012), it remains uncertain whether this is comparable with other countries. Therefore, this study focuses on the Netherlands as a different setting. Furthermore, to the best of our knowledge, no studies have used a qualitative approach with the aim of gaining in-depth insights into the perspectives of clients. Therefore, the aim was to understand the suitability of smoking cessation support provided by SCSOs, from the perspective of people with a low SEP, who smoke or have smoked regularly. Specifically, our objectives were to (i) investigate the suitability of smoking cessation support provided by SCSOs, and (ii) explore their reasons for considering it suitable or unsuitable.

## METHODS

### Design

We conducted a qualitative study consisting of 19 semi-structured interviews with individuals who are currently smoking or have smoked in the past. The semi-structured format can produce rich data that provide insights into participants’ experiences and perceptions (Peters and Halcomb, 2015). This study took place in the North district of Amsterdam, the Netherlands, which is characterized by a relatively high prevalence of smoking and a relatively low SEP (Gradener *et al*., 2021; Hoedemaker *et al*., 2023). In this district, several smoking cessation support activities are already being undertaken within SCSOs which makes the setting more suitable. However, practices are not yet considered standard across the Netherlands (Visser *et al*., 2024).

### Sample and recruitment

To be eligible to participate, individuals had to fulfill two of the three criteria for low SEP, which included educational attainment (secondary vocational education or lower), employment status (unemployed) and financial status (experiencing financial difficulties or great financial difficulties) (Vidrine *et al*., 2009). In addition, participants had to live in Amsterdam North and were included only if they had interacted with at least one SCSO. Furthermore, participants needed to smoke regularly or have smoked regularly in the past. Lastly, participants were required to possess a sufficient level of proficiency in the Dutch language. Given our strategy to invite participants through SCSO professionals (see recruitment strategy below) smoking status was known at the time of recruitment, and this was verified in the questionnaire that participants filled in (see description under ‘Instruments’).

Convenience sampling was used to recruit the participants. We reached out by telephone to SCSO professionals based in Amsterdam North, with whom we already had existing contacts. We asked them for their assistance in recruiting participants, and for permission to visit smoking cessation groups in SCSOs to recruit participants ourselves. Initially, participant recruitment involved visiting two smoking cessation groups located within SCSOs in Amsterdam North. Information about the study was provided to participants of these groups. Next, interested participants were invited for the study (*N* = 6). The remaining participants (*N* = 15) were recruited through face-to-face interactions with SCSO professionals working in Amsterdam North with whom prior contact had been established; however, two participants were found to be ineligible to participate. After recruitment by the SCSO professionals, the researcher contacted the participants by telephone to schedule the interview.

Data collection in this study followed the principle of data saturation, which was achieved after the 14th interview. To ensure completeness, the researchers conducted an additional five interviews to verify that no data was left out.

### Procedure

The data collection took place between August 2022 and January 2023. Interviews were conducted by the first author (J.V.), a female researcher trained in qualitative methods who has previously published on qualitative research (Visser *et al*., 2024). Interviews were conducted in a private setting, either at the respective SCSO or at the participant’s home. The participants were provided with an information letter that included all necessary details regarding the study purpose, data use and informed consent. Comprehension was ensured by verbally explaining this information, inviting participants to ask questions and obtaining verbal consent (Tekola *et al*., 2009). Interviews lasted 37 min on average (range 16–55). Before the interview, each participant completed a short questionnaire with demographic questions. After the interview, participants were offered a voucher of 20 euros. The interviews were audio-recorded and transcribed verbatim. The study was guided by qualitative description methodology and reported using the consolidated criteria for reporting qualitative research (COREQ) (Supplementary Material S1) (Tong *et al*., 2007).

### Instruments

The questionnaire assessed demographic variables, including gender, age, country of origin, educational attainment, employment status, financial status, living conditions and smoking status.

By centering the interview guide around participants’ experiences and needs, we ensured that the questions were accessible and understandable for the target audience (Supplementary Material S2). In the first part of the interview guide participants were asked about their experiences with smoking cessation support provided by SCSOs. This section was designed to encourage participants to share their stories in an accessible manner, which in turn revealed which aspects make SCSOs suitable or unsuitable for providing support. In the second part of the interview guide participants were asked about their needs of smoking cessation support from SCSOs and the underlying motives of these needs. This section was designed to encourage participants to share their needs for smoking cessation support and to investigate the suitability of SCSOs to meet these needs.

In case the participants experienced difficulties in understanding or answering the questions, the interviewer rephrased the question or provided additional explanations.

### Data analysis

A short questionnaire containing demographic questions was used to describe the study population. Interview transcripts were analyzed following the thematic approach of Braun and Clarke in MAXQDA 2022 (Braun and Clarke, 2006). An inductive approach was used to develop codes, sub-themes and themes (Azungah, 2018).

First, the first and second authors (J.V. and J.B.) became familiar with the data by reading and re-reading transcripts, listening to audio recordings and noting any initial observations. Next, one transcript was independently open-coded by these authors. Inconsistencies in coding were discussed to ensure agreement on coding principles. The double-coding process continued until the sixth transcripts, when full consistency in coding was reached. The remaining transcripts were independently coded by the second author (J.B.), as there was consistency with the previous parallel-coded transcripts. In case she had doubts during the coding process, the first author (J.V.) was consulted. Subsequently, different codes were combined to form overarching sub-themes, which were categorized into five main themes (location, setting, the professional, the support and additional activities alongside support). This process of combining codes into overarching themes was iteratively checked, reviewed and refined by different authors (M.K., J.V. and J.B.) through multiple discussions. In addition, the themes were reviewed with a member-checking focus group, consisting of six participants, to see how well the themes resonated with participants’ experiences and perceptions (Birt *et al*., 2016). Lastly, agreement was reached on the theme names and content, which formed the basis for the write-up of findings (presented below).

## RESULTS

The background characteristics of the participants are presented in Table 1. Among the participants, there were slightly more women than men, with the majority of the participants being over the age of 50. The majority of participants had lower levels of education, were unemployed, or experienced at least some financial difficulties. A minority of participants had previously participated in a smoking cessation program within an SCSO.

**Table 1:** Characteristics of interviewed participants (*n* = 19)

	*N*	%
Total	19	100
Gender		
Male	9	47
Female	10	53
Age		
18–29	1	5
30–39	2	11
40–49	4	21
50–59	7	37
60–69	5	26
Smoking status		
Current smoker	14	74
Past smoker	5	26
Country of origin		
Netherlands	13	68
Other	6	32
Education		
No education	3	16
High school	8	42
Secondary vocational education (MBO)	5	26
Higher vocational education (HBO)	3	16
Employment		
Unable/incapacitated to work	11	58
Unemployed/seeking work	4	21
Paid employment	3	16
Retired	1	5
Financial difficulties		
Yes, great difficulties	3	16
Yes, some difficulties	9	47
No difficulties, but necessary to watch expenses	6	32
No difficulties	1	5
Cessation program within SCSOs		
Yes	7	37
No	12	63

### Suitability of smoking cessation support provided by SCSOs

Participants indicated that SCSOs may be suitable for providing support in smoking cessation. ‘*Yes, it would be nice if there is a possibility here in the community center’ (Female, aged 50–59, current smoker). ‘I think that could have quite an impact [smoking cessation support from SCSOs]’ (Female, aged 18–29, current smoker).* Some participants mentioned that they did not seek any type of support for smoking cessation, either believing it should come from personal willpower or because they were dealing with stressors, making it challenging to prioritize quitting smoking. *‘I have a lot of stress in my head, and when I don’t feel good, I’m quickly tempted to reach for a cigarette to relieve the stress, which is actually nonsense, but that’s how it is, and for me... I don’t think it works [smoking cessation support]’ (Male, aged 50–59, current smoker).* However, if these participants were to seek support, they considered SCSOs to be suitable as well. *‘I think social workers, you know, those kinds of people in your environment, would be the ones you would approach [to ask for help]. Not that you walk into a hospital or something and say, “I want to quit”’ (Female, aged 50–59, former smoker).* While participants generally believe that SCSOs would be suitable, they also mentioned reasons why it might be less suitable. These are discussed below.

### Reasons for suitability or unsuitability

Participants mentioned several reasons that would make SCSOs either suitable or unsuitable for providing smoking cessation support.

#### Location

Participants consider SCSOs as a suitable location for obtaining smoking cessation support. First, SCSOs are located within walking or cycling distance from their homes. This serves as a facilitating factor for participation because it removes difficulties in reaching these organizations. For example, it prevents financial barriers such as paying for public transportation or parking and fuel costs. Second, participants mentioned that they are familiar with the locations of SCSOs, which results in them feeling comfortable and safe to access these organizations. *‘I would prefer to do it [smoking cessation support] here [the community centre], because I’m not the type to go to somewhere unfamiliar. Maybe that doesn’t apply to someone else, but for me, this is low-pressure, it is relaxed, it is close by, at least I’ll make use of it in this way’ (Female, aged 60–69, current smoker).*

#### Setting

Participants experienced that SCSOs offer the flexibility of receiving support in both group and individual settings or a combination of both. This allows participants to choose a setting that aligns with their specific needs. Some expressed a preference for a group setting as it provides an opportunity to exchange experiences and give each other advice, fosters a sense of community with a common goal and gives them a sense of not being alone. Participants expressed a positive attitude toward support in a group setting within SCSOs as they perceived it as enjoyable, motivating and accessible, as well as supportive. *‘People struggle with the language barrier. In a group setting, you can help translate a bit’ (Male, aged 40–49, former smoker). ‘Some go to the GP, and that can be difficult. They give you five minutes or three minutes. The GP provides you stickers [nicotine patches]. You can also buy that at [a Dutch shop]. But when you have discussions here [in SCSOs groups] with an entire group, we help each other’ (Male, aged 40–49, former smoker).* Individuals also expressed a preference for an individual setting within SCSOs as they feel more comfortable being vulnerable in a one-on-one session and are more willing to share individual issues, which they might not express as readily or perhaps as casually in a group setting. Participants have learned from previous experiences that individual support from SCSO professionals has made them feel at ease to shed tears.

Participants varied in their opinions regarding the timing of support provided by SCSOs. For example, participants experienced having to wait for support after registration, which is unfavorable. *‘If it takes a month or two or three [before support starts] then I no longer have any motivation. The motivation is long gone’ (Female, aged 40–49, former smoker).* In contrast, other participants mentioned SCSOs do arrange support quickly after a request for help.

#### The professional

Participants emphasized that SCSO professionals possess characteristics that make them well-suited to provide smoking cessation support. Participants usually already know the SCSO professional and have built a relationship of trust with them. This makes the professional approachable, participants know what to expect from the professional and have the confidence that they will receive help from someone who already genuinely supports them. *‘I would prefer that [support from a SCSO professional]. Those people know more about you, so I think they can guide you better than the GP’ (Male, aged 50–59, current smoker).*

Furthermore, they have previously experienced that SCSO professionals demonstrate involvement and provide consistent support. *‘You can’t speak to the GP during the day and night, but with the ladies from [a community center], it’s easier’ (Female, aged 40–49, current smoker). ‘They are all caring people, and just knowing that they are there, and that if you have problems, you have somewhere to go to’ (Male, aged 30–39, current smoker).* This could encourage participants to continue with their efforts to quit smoking and prevent them from giving up quickly. *‘If you didn’t attend [at an activity organized by SCSO], they [SCSO professionals] would call you and ask how you are doing, or they would send a message. They can’t do everything to help you. You also have to do it yourself, of course. But it does work. Then I’m going for it again’ (Male, aged 50–59, current smoker).*

Participants indicated the importance of a professional with the necessary expertise to offer smoking cessation support and they mentioned that the needed support might exceed the expertise of the SCSO professional. Nevertheless, they are confident that the professional would find a more specialized professional to refer to. However, participants argued that there must be a connection between the client and the person to whom the client is referred, and the support is provided within the local community. They stressed that people may feel uncomfortable with unfamiliar professionals in distant settings. *‘I don’t think that would be wrong in itself [SCSO professionals referring to smoking cessation services], but then the same question remains: “Would that connection be there?” What is also important to me is that you are not referred from this side to the other side of Amsterdam North or to Amsterdam West, which requires either public transport or a car, resulting in paid parking costs and additional fuel expenses’ (Female, aged 40–49, smoker).*

#### The support

Participants believe that SCSOs would provide personalized support tailored to the individual needs, which, according to the participants, is essential. This implies that a professional assesses what is needed for each individual to engage in a successful quitting process as the quitting process varies for each participant. *‘They [SCSO professionals] really delve into: “who are you and what can we do for you?” It’s not like they always do the same thing, but they really look at the individual: what works for you? And that’s very good’ (Female, aged 18–29, current smoker).*

In addition, participants consider SCSO support suitable as they take into account the individuals’ context and other issues, not just smoking, which means that they work in a holistic manner. Participants deal with a combination of problems, with many co-occurring and interacting factors. For instance, the stress resulting from feelings of loneliness may lead to smoking, or quitting smoking may lead to challenges in managing weight gain through nutrition. They mentioned the importance of taking into account the factors that are related to their smoking behavior, such as feelings of loneliness. *‘They [SCSO professionals] think with you: “yes, you’ve gained some weight. Let’s incorporate exercise.” The first time I went [to an exercise group] on Wednesday afternoon, [SCSO professional] went with me’ (Female, aged 40–49, current smoker).*

Nonetheless, there are some remarks. Clients highlighted the importance of aftercare (i.e. support received after smoking cessation treatment, primarily aimed at preventing relapse) with a gradually decreasing frequency of contact. This could enhance their confidence in successfully remaining abstinent. However, according to participants, aftercare did not appear to be a standard practice within SCSOs. *‘The ability to fall back on someone you already know, who is aware of the situation and can easily intervene. I think it is disappointing that you don’t have that kind of support anymore’ (Male, aged 50–59, current smoker).* Although this may not be standard practice, participants indicated that they have experience with aftercare from SCSOs. *‘It is nice that such a group exists [walk-in group organized by SCSO organization], where you can return during challenging moments and receive attention when needed’ (Female, aged 60–69, former smoker).*

#### Additional activities alongside support

According to the participants, SCSOs offer additional activities alongside smoking cessation support, which could contribute to their smoking cessation process. They indicated that it is standard practice for SCSOs to, for example, organize walking groups or facilitate volunteer work. These activities play a vital role in facilitating a successful quit attempt by positively enhancing both the physical and mental well-being of the participants. In addition, these activities could serve as effective distractions during challenging moments. *‘They [SCSO organization] have those sports groups. It’s a few hours a week, I believe. You are active, so you don’t think about it so much that you get a craving for a cigarette’ (Female, aged 18–29, current smoker).* Moreover, people are surrounded by others when engaging in SCSOs group activities, making them less likely to smoke.

## DISCUSSION

### Key findings

The study aimed to investigate the suitability of smoking cessation support provided by SCSOs, according to people with a low SEP, who smoke or have smoked regularly, and to explore their reasons for considering it suitable or unsuitable. Participants indicated various reasons for SCSOs being suitable for providing smoking cessation support. These factors related to the location (i.e. nearby and familiar locations), setting in which SCSOs operate (i.e. individual and group setting), SCSO professionals (i.e. involved, relationship of trust), type of support offered (i.e. personalized support, holistic approach) and additional activities provided (i.e. facilitating walking groups and volunteer work). Factors that make SCSOs less suitable for providing smoking cessation support related to the waiting time for support, professionals’ limited expertise and the limited provision of aftercare to prevent relapse.

### Interpretation of the findings

In this study, participants emphasize the importance of considering the broader context of an individual, rather than solely focusing on smoking cessation. This aligns with findings from a systematic review exploring perceived barriers to smoking cessation in selected vulnerable groups (Twyman *et al*., 2014). The review revealed that smoking is used as a coping mechanism in response to daily stressors as well as the stress inherent in vulnerable lives. Focusing solely on smoking cessation may not be sufficient, as it does not address the underlying issues related to clients’ smoking behavior. Interestingly, standard cessation care, for example, from the general practitioner or practice nurse, primarily concentrates on nicotine addiction, overlooking the impact of social circumstances on smoking behavior and relapse (Zwar *et al*., 2006; de Ruijter *et al*., 2017; van Wijk *et al*., 2019). A randomized controlled trial demonstrated that incorporating a program focused on enhancing their coping skills for anger and stress, in addition to standard smoking cessation care, led to a higher quit rate after 6 months compared to receiving standard cessation care alone (Yalcin *et al*., 2014). Another study demonstrated that exercise enhances the likelihood of successful quitting for smokers with high levels of anxiety sensitivity (Smits *et al*., 2016). This highlights the importance of holistic support and the potential of SCSOs, incorporating elements that address the underlying issues, particularly for people with lower SEP (van Wijk *et al*., 2019).

The results of this study also show that the needed support might exceed the expertise of SCSO professionals. This is consistent with previous studies showing that SCSO professionals may not have sufficient skills and knowledge to address smoking issues among their clients (Bryant *et al*., 2010; Hull *et al*., 2012; Visser *et al*., 2024). This may be because smoking cessation support is not included in the training or education of professionals (Bryant *et al*., 2010; O’brien *et al*., 2012; Kleinfelder *et al*., 2013). Smoking cessation training could be useful to improve professionals’ knowledge, confidence and skills, thereby increasing their likelihood to provide smoking cessation support (O’Brien *et al*., 2010; Bryant *et al*., 2012; O’brien *et al*., 2012). One study showed that, as a result of smoking cessation training among SCSO professionals, clients significantly reduced the number of cigarettes smoked, though none of them reported abstinence at 6-month follow up (Bryant *et al*., 2012). Training may be beneficial, but it does not directly lead to increased cessation success. Achieving higher cessation success may require more than just training.

The study reveals that clients are satisfied with professionals’ involvement and consistent support, which aligns with earlier studies indicating that professionals from SCSOs have regular and often long-term contact with clients (Bryant *et al*., 2012; Bonevski *et al*., 2013). Standard smoking cessation care is often insufficiently intensive for low SEP people (van Wijk *et al*., 2019; Troelstra *et al*., 2021). For example, interventions commonly end after a small number of sessions or after a few months without a follow-up moment (Troelstra *et al*., 2021). Research showed that individuals with lower SEP need additional support between sessions, one year of follow-up support, and a procedure to prevent relapse (van Wijk *et al*., 2019; Troelstra *et al*., 2021). This could be explained by previous findings indicating that, on average, people from lower SEP groups tend to be more nicotine dependent, receive less social support, and experience greater stress due to, for example, financial uncertainty, challenging working conditions and inadequate housing (van Wijk *et al*., 2019; Troelstra *et al*., 2021). The support provided by SCSO professionals may be beneficial in fulfilling the needs of clients. However, SCSO professionals are reported to be ‘overloaded’ with their current caseloads (Bryant *et al*., 2010), which raises concerns about the intensity of support they can actually provide. Prerequisites are that government and organizations prioritize smoking cessation and allocate sufficient time for professionals to address smoking with their clients (Visser *et al*., 2024).

### Strengths and limitations

This was, to our knowledge, the first study to use qualitative methods to explore the perceptions of people from lower SEP groups regarding smoking cessation support provided by SCSOs. This approach is important for gaining a deeper understanding of the potential of SCSOs to provide smoking cessation support, according to the group receiving the support. Second, a strength of the study is that we conducted a member-check focus group, which confirmed that the analysis aligned with participants’ perspectives, thereby enhancing the trustworthiness of the results (Birt *et al*., 2016).

This study also has a number of potential limitations that should be considered when interpreting the data. First, participants may have provided socially acceptable answers as they may be concerned about the possibility of the data being shared with their SCSO professionals, despite the assurance that the data would be evaluated and reported pseudonymously. However, given that participants highlighted various reasons for SCSOs being perceived as less suitable, we do not consider social desirability to have played a major role. Second, a relatively small sample size is used in this study; however, the goal of qualitative research is to gain insight in participants’ experiences and perspectives (Marshall, 1996). Therefore, our focus is on achieving depth of understanding rather than breadth of information and generalizability. We can confidently assert that this goal has been achieved, as data saturation was reached. Third, clients’ perspectives are shaped by experiences with SCSOs in a specific neighborhood of Amsterdam. Even though saturation was achieved, it is conceivable that SCSOs elsewhere operate differently, potentially reducing the wider applicability of the results.

### Implications

This study has several implications for policy, practice and research. SCSO professionals’ knowledge and skills in providing smoking cessation support could be enhanced through education and training. The topic of smoking cessation may be incorporated into social work education programs. In addition, SCSO professionals may receive training to enhance their expertise in providing smoking cessation support. However, to facilitate smoking cessation in SCSOs, policy changes within and outside SCSOs are needed. For example, prioritization of health and smoking in SCSO organizations enables funding for training programs and allows professionals to incorporate smoking cessation in their work, and creating smoke-free environments around SCSOs supports those who try to quit (Frazer *et al*., 2016; Visser *et al*., 2024).

Further research should investigate the possibilities of training and education for professionals in providing smoking cessation support, and the effectiveness of smoking cessation support provided by SCSOs in achieving smoking cessation among clients. Given professionals’ potential workload, it is also crucial to explore how smoking cessation support could be integrated into their work tasks.

## CONCLUSION

According to people with a low SEP who smoke or have smoked regularly, SCSOs could be well-suited to provide support for smoking cessation, although they may not be able to meet all needs for optimal support. Support from SCSOs can be an important complementary opportunity for people with low SEP in addition to standard smoking care, because they may be perceived as more accessible and could be able to address both the smoking behavior as well as the underlying problems of clients’ smoking behavior. However, clients have doubts about the capability of SCSOs and the professionals working within these organizations to provide effective support. To harness the potential of SCSOs, smoking cessation could be integrated into educational programs and training for professionals could be promoted. Policy changes would be needed to support these efforts.

## Supplementary Material

daae141_suppl_Supplementary_Materials_1

daae141_suppl_Supplementary_Materials_2

## Data Availability

Interview transcripts cannot be shared publicly because of ethical restrictions.
