# Supplementary material for: Suitability of smoking cessation support from social and community service organizations: perspectives of Dutch clients
Source: Health Promot Int. 2024 Oct 24;39(5):daae141. doi: 10.1093/heapro/daae141 (PMC11500714; doi:10.1093/heapro/daae141)
Supplement: daae141_suppl_Supplementary_Materials_2 [file daae141_suppl_supplementary_materials_2.docx]

**Appendices**

APPENDIX 1. Translated interview guide

| **Introduction interview: information and questions** *(duration approximately 5 minutes)* | |
| --- | --- |
| **Introduction** | - Glad you would like to participate in this study and make time for it - Introduce yourself: 'I am working as a researcher, conducting this research commissioned by Amsterdam UMC in collaboration with Tranzo, Tilburg University.' - Stress confidentiality of the research! |
| **Explanation of the aim** | - Little is yet known about the support clients would like to receive from SCSOs in quitting smoking and why. In this interview we will discuss your experiences with smoking cessation support from these organizations and the support you would like to receive from them to quit smoking or to stay quit and the reasons for this. In this way we can explore why and how these organizations can support you quit smoking. |
| **Participants** | - Participants will be interviewed between August 2022 and January 2023. |
| **Topics** | The following topics are discussed in the interview:   - The experiences of smoking cessation support from SCSOs - The needs for smoking cessation support from SCSOs |
| **Duration interview** | - Approximately between 30 and 45 minutes |
| **Ending of introduction** | - If questions are not clear, please let us know. - Do you have any questions for now? |
| **Confidentiality** | - Requesting permission to make audio recordings so that we can fully elaborate on the data at a later time. These recordings will be kept in a secure environment within the Amsterdam UMC, location AMC. The data from the interviews will then be analyzed pseudonymously - Ask permission to use anonymized statements for scientific articles. - Answers are not right or wrong, sharing from own experience and opinion. - We are not affiliated with the municipality, and we will not share this information with the municipality or any other organizations." - Recorder on - Ask respondent for permission/informed consent when recorder is on, e.g., 'Can you confirm that you would like to participate in the study and consent to the recording of this interview?' Respondent's response: 'Yes' |

| **Core of the interview** | | |
| --- | --- | --- |
| **Start** | We will now start the interview | |
| 1. We start with your experiences regarding smoking cessation support in the neighborhood | **The experiences of smoking cessation support from SCSOs**   - To what extent do you have contact with organizations in the neighborhood that offer support? - If not clear, for example, assistance with financial matters, job searching, your residence... - If not clear, for example, a budget coach, a social worker... - With whom? - What has [professional SCSO] discussed with you regarding smoking/quitting smoking/smoking cessation support? - Can you describe how this conversation went? - How did you experienced this? - What happened afterward? - And how did you experienced that? - How has [professional SCSO] supported you in quitting smoking? - What did this support entail? - Optional: What is the reason that [professional SCSO] did not support you in quitting smoking? - What were your experiences with the support? - What worked well? Why? - What worked less well? Why? - In what way did this support differ from other smoking cessation support you have experienced in the past? - To what extent did one type of support have your preference? - What are the reasons for your preference?   *If the participant indicates never having discussed smoking/quitting smoking support with the SCSO professional:*   - Why has it never been discussed? - What are your thoughts on this? Why?   *Prompts for the type of support (let the participant speak first, then provide options):   - Ask about smoking status. - Shared consequences of smoking. - Advice to quit smoking. - Increased motivation to quit smoking. - Smoking cessation support program offered by the professional. - Referral to the general practitioner. - Referral to internal/external smoking cessation support programs. - Prevention of relapse. - Information session on quitting smoking. - Connected with experienced individuals.   *Interim and concluding summaries; ask: did I understand correctly that...? (Duration approximately 15 minutes)* | |
| 1. We now delve into the needs regarding smoking cessation support from SCSOs | **The needs for smoking cessation support from SCSOs**   - In what way would you like to be approached regarding smoking/cessation support from [SCSOs]? - By whom? - Why? - What support would help you well/what support would help you less? - If you were to attempt quitting smoking again, in what way could [SCSOs] support you? - By whom? - Why? - What support would help you well/what support would help you less?   *You mentioned this type of support earlier, and you expressed the following thoughts about it...*   - What other support would you like to receive from [SCSOs]? - By whom? - Why? - What support would help you well/what support would help you less?   *Suppose you would like to make another attempt to quit smoking, how would you feel if [professional SCSO]...*   - ...initiates a conversation about smoking and asks you about your thoughts on quitting smoking. - ...advises you to quit smoking. - ...tries to increase your motivation to quit smoking. - ...refers you to an internal or external smoking cessation program. - ...refers you to the general practitioner to quit smoking. - ...personally guides you in quitting smoking. - ...connects you with an experienced individual.   If you could choose any smoking cessation support, what type of support do you prefer? (Support not necessarily from SCSOs)   - By whom? - Why?   *Intermediate and concluding summaries; ask: have I understood correctly that...? (Duration approximately 20 minutes)* | |
| **Completion of interview** (Duration about 5 minutes) | | |
| **Closing interview** | | - Is there anything else you would like to add to the conversation? - Are there things not yet covered that you feel are important to this study? - What did you think of the interview? |
| **Thanking for interview** | | - I would like to thank you for the interview and the time you were willing to give |
| **Additional questions** | | - If there are any questions, please contact me |
| **Stop recording** | |  |
